# Role and Therapeutic Targeting of the HGF/MET Pathway in Glioblastoma

**DOI:** 10.3390/cancers9070087

**Published:** 2017-07-11

**Authors:** Nichola Cruickshanks, Ying Zhang, Fang Yuan, Mary Pahuski, Myron Gibert, Roger Abounader

**Affiliations:** 1 Department of Microbiology, Immunology & Cancer Biology, University of Virginia, Charlottesville, VA 22908, USA; nac5t@virginia.edu (N.C.); yz5h@virginia.edu (Y.Z.); fy5dm@virginia.edu (F.Y.); mary.pahuski@gmail.com (M.P.); mkg7x@virginia.edu (M.G.); 2 Department of Neurology and The Cancer Center, Department of Microbiology, Immunology & Cancer Biology, University of Virginia, Charlottesville, VA 22908, USA

**Keywords:** Glioblastoma, HGF, MET

## Abstract

Glioblastoma (GBM) is a lethal brain tumor with dismal prognosis. Current therapeutic options, consisting of surgery, chemotherapy and radiation, have only served to marginally increase patient survival. Receptor tyrosine kinases (RTKs) are dysregulated in approximately 90% of GBM; attributed to this, research has focused on inhibiting RTKs as a novel and effective therapy for GBM. Overexpression of RTK mesenchymal epithelial transition (MET), and its ligand, hepatocyte growth factor (HGF), in GBM highlights a promising new therapeutic target. This review will discuss the role of MET in cell cycle regulation, cell proliferation, evasion of apoptosis, cell migration and invasion, angiogenesis and therapeutic resistance in GBM. It will also discuss the modes of deregulation of HGF/MET and their regulation by microRNAs. As the HGF/MET pathway is a vital regulator of multiple pro-survival pathways, efforts and strategies for its exploitation for GBM therapy are also described.

## 1. Glioblastoma

Glioblastoma (GBM) is the most common and lethal primary malignant brain tumor with an incidence rate of approximately 3.19/100,000 per year [1]. Despite intensive therapy including surgical resection, chemotherapy and radiotherapy, prognosis remains poor with an average survival of 14 months [2]. The Cancer Genome Atlas Research Network (TCGA) identified genomic alterations present in GBM, classifying the tumors into four distinct subtypes: classical, proneural, mesenchymal and neural [3]. Characterized by overexpression of epidermal growth factor receptor (EGFR), classical GBM often lack TP53 mutation and display focal loss of 9p21.3. The majority of proneural GBM, on the other hand, harbor TP53 mutations (over 50%) along with mutations of the isocitrate dehydrogenase 1 gene (IDH1). In the mesenchymal subgroup, deletion of region 17q11.2, correlating to neurofibromatosis gene (NF1), is the most frequent alteration, followed by mutations in phosphatase and tensin homolog (PTEN), TP53 and mesenchymal epithelial transition (MET) overexpression. The neural subset of GBM encompassed mutations described in other subtypes but displayed no subtype specific mutations; additionally, this group of patients tended to be older [4]. Recently, the neural subtype of GBM has been called into question and is thought to represent normal brain contamination. Additionally, GBMs are further categorized based on the World Health Organization (WHO) classification. This updated classification separates central nervous system (CNS) tumors based on cell origin, grade, molecular alterations, such as IDH-mutation, and histology [5]. Three core pathways were found to be almost universally deregulated in GBM: the p53 (87%), retinoblastoma (RB) (78%) and receptor tyrosine kinase (RTK) (88%) signaling pathways. Within the p53 pathway dysregulation of GBM, homozygous deletion of p14/ARF is the predominate alteration, followed closely by mutation or deletion of the tumor suppressor (TP53) itself. Deletion or mutation of p16 and CDKN2B in the RB pathway was identified as another common alteration displayed by GBM. The most frequent RTK alteration identified remains amplification or mutation of EGFR (45%) followed by epidermal growth factor receptor (ERBB2) (8%), platelet-derived growth factor receptor α (PDGFRα) (13%) and MET (4%). Other common alterations in this pathway include mutation or deletion of tumor suppressors PTEN and NF1 [6,7].

Given the dismal prognosis of GBM, research has focused on identifying novel targets for therapy. A particular area of interest are RTKs that regulate many essential cellular processes within normal cells, such as cell proliferation, differentiation and survival [8,9]. Deregulation of RTKs is common in the initiation and progression of GBM, as highlighted by TCGA analysis, emphasizing their potential as targets for new anticancer therapies.

### 1.1. HGF and MET

The RTK MET is coded for by the MET proto-oncogene located on chromosome 7q21–31 [10,11]. MET is spontaneously deregulated in approximately 2–3% of cancers [12] and activated primarily in the mesenchymal high-grade subtype of GBM [13,14]. MET regulates multiple cellular functions such as proliferation, survival and motility and displays low activity in normal cells. Aberrant MET activation in tumor cells promotes enhanced tumor cell growth, angiogenesis and invasion and is associated with poorer overall survival [8,15,16]. Oncogenic MET activation can result from various mechanisms including amplification of MET, elevated levels of its ligand, hepatocyte growth factor (HGF), mutations within the promoter region of HGF, constitutive kinase activity due to mutation and loss of negative regulatory mechanisms such as microRNAs [10,15,17,18]. Since the MET pathway is predominately activated in high-grade GBM cells, targeting MET could lead to selective killing of tumor cells whilst sparing normal cells for optimal anticancer therapy [15].

**Figure 1:**
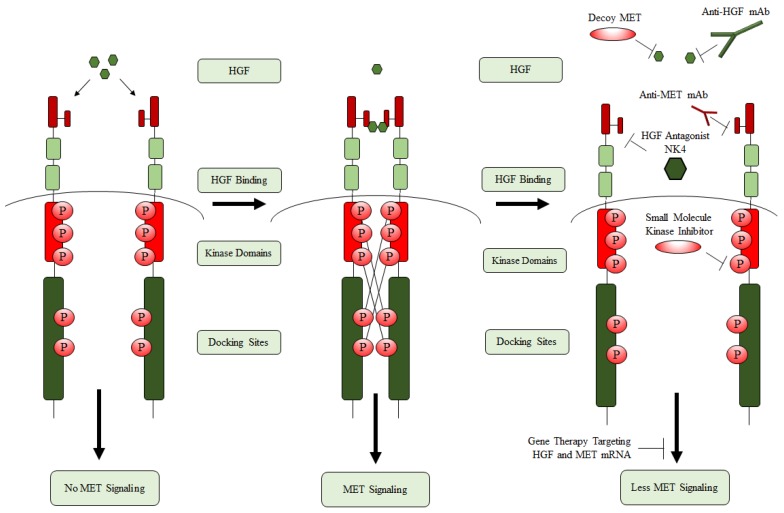
Mechanism of mesenchymal epithelial transition (MET) activation and current therapeutic strategies for hepatocyte growth factor (HGF)/MET inhibition. The MET receptor is activated through ligand (HGF) binding, which induces receptor dimerization and cross phosphorylation. Activation can be inhibited in several ways: (1) Decoy MET protein sequesters HGF from MET, (2) Anti-HGF and MET monoclonal antibodies competitively bind to the ligand and receptor, respectively. (3) HGF antagonists competitively bind to MET. (4) Small molecule kinase inhibitors prevent receptor activation by inhibiting kinase domain activity. (5) Gene therapy modulates HGF and MET mRNA production.

The MET receptor is a dimeric, 190 kD tyrosine receptor kinase expressed on the surface of epithelial and endothelial cells and at low levels in the brain. The dimer features a 50 kD extracellular α-chain and a 140 kD transmembrane β-chain, linked together by disulfide bridges. The only known ligand for MET is hepatocyte growth factor (HGF) (also known as the scatter factor, SF), which is a multifunctional two-chain cytokine secreted by mesenchymal cells (Figure 1) [10,19]. HGF is initially synthesized as pro-HGF which is then cleaved into mature HGF [20]. Mature HGF consists of six protein domains: N-terminal domain, four kringle domains and a C-terminal domain [11,21]. When mature HGF binds to MET it sits within the ligand-binding pocket of the extracellular α-chain of MET, while the intracellular β-chain of MET contains the tyrosine kinase domain [16,22]. Binding of HGF to MET results in receptor dimerization and the autophosphorylation of multiple tyrosine residues—Y1234 and Y1235 responsible for receptor activity, and Y1349 and Y1356 responsible for the recruitment of downstream effectors [15,19]. The latter enables the binding of a variety of substrates to MET which in turn leads to the activation of multiple signaling pathways [10].

### 1.2. HGF/MET Downstream Signaling

**Figure 2:**
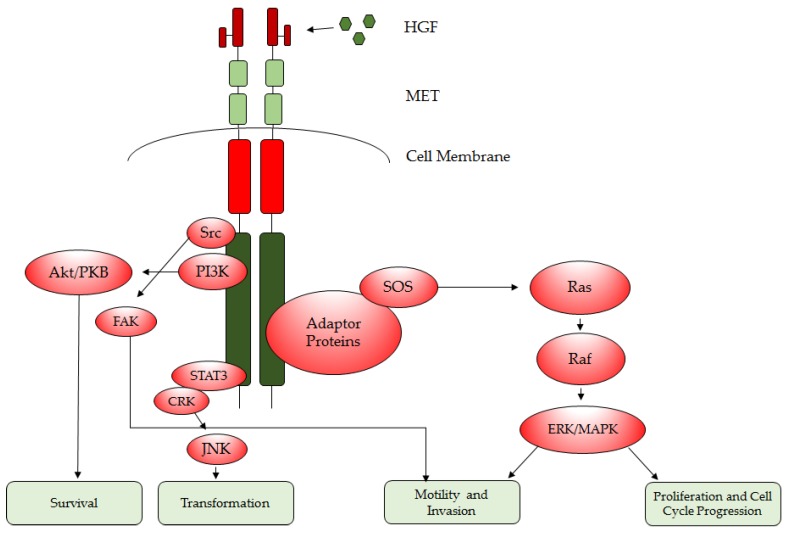
MET regulated signaling pathways implicated in the hallmarks of cancer. MET activation initiates the following downstream pathways: (1) phosphoinositide 3-kinase (PI3K) signaling conferring enhanced survival, (2) RAS/mitogen-activated protein kinase (MAPK) Pathway signaling resulting in enhanced proliferation, cell motility and invasion, (3) Jun amino-terminal kinases (JNK)/signal transducer and activator of transcription (STAT) signaling, which contributes to cell transformation and (4) focal adhesion kinase (FAK) signaling leading to increased cell motility and invasion.

The oncogenic effect of MET is mediated through the recruitment of multiple effectors including GRB2, GAB1, PI3K, Shc, Src and SHP-2, ultimately leading to the activation of several downstream signal transduction pathways (Figure 2) [8,14,16]. One such pathway is the mitogen-activated protein kinase (MAPK, a serine/threonine kinase) network, which is stimulated through GRB2-SOS and MET association and subsequently RAS activation. The MAPK signaling cascade includes extracellular signal-related kinase (ERK1/2), Jun amino-terminal kinases (JNK1/2/3), p38-MAPK and ERK5 and is vital in the regulation of cell proliferation, survival and differentiation [10]. Another effector mediated by MET is phosphoinositide 3-kinase (PI3K), which is activated either through direct binding of the p85 subunit to MET or indirectly through RAS recruitment causing subsequent phosphorylation of AKT. Like its counterpart, the MAPK cascade, this pathway is critical in many cell regulatory functions such as growth, proliferation and survival leading to the promotion of tumorigenesis [10,23]. Further downstream from traditional kinase activation routes, MET activation also leads to phosphorylation of the Janus kinase signal transducer and activator of transcription (STAT), which dissociates from the receptor and relocates to the nucleus to promote tumor cell proliferation, survival and invasion [10]. MET’s role in mediating these pathways ultimately makes it a centralized regulatory node of many pro-survival signaling networks, many of which are often exploited by transformed cells (Figure 2).

### 1.3. HGF/MET Regulation by microRNAs

MicroRNAs (miRNAs) provide a novel layer of signaling regulation outside of the traditional central dogma. miRNAs are non-protein-coding RNAs that bind their mRNA targets through imperfect base pairing. Once bound, miRNAs function by either inhibiting the translation of their mRNA targets or promoting their degradation [24]. A subset of miRNAs have been shown to specifically regulate the signaling output of the MET pathway. Conversely, the MET pathway has been shown to regulate the expression of another subset of miRNAs to provide feedback on itself. Dysregulation of either has been implicated in cancer development.

miR-410 [25], miR-144-3p [26] and miR-449b-5p [27] all inhibit GBM growth mainly by targeting the translation of the MET protein. RNA profiling and FISH show reduced expression of these miRNAs in human GBM samples compared to normal brain samples. Furthermore, their expression inversely correlates with that of MET. GBM cells transfected with either miR-410 or miR-144-3p exhibit reduced proliferation (in vitro and also in a xenograft model), reduced cell cycle progression, reduced invasion, and increased apoptosis. Such phenotypes are rescued when the cells are co-transfected with MET. Interestingly, miR-449b-5p is further regulated by NEAT1 through sequestration, and NEAT1 is highly expressed in high-grade GBM samples, providing a novel regulatory mechanism of miRNAs.

Two other important miRNAs are miR-34a [28] and miR-182 [29]. While these miRNAs have been shown to influence the expression of MET, they are coincidentally implicated in the regulation of other pathways. miR-34a also targets NOTCH, EGFR, and platelet-derived growth factor receptor α precursor (PDGFRA), all of which are key players in GBM development. Interestingly, miR-34a itself is induced by wildtype P53 but not by mutant P53. A model is proposed where wildtype P53 regulates GBM growth by inhibiting the MET and the NOTCH pathway via miR-34a. Such regulation is lost in the presence of mutant P53. On the other hand, miR-182 is shown to inhibit proliferation, induce apoptosis and reduce the stem cell-like characteristics of GBM cells by targeting MET, BCL2L21 and HIF2 respectively.

Conversely, miRNAs also participate in a feedback loop within the MET and other RTK pathways. RTKs repress the transcription of these miRNAs, which usually repress the RTK signaling, thus forming a positive feedback loop; an example is miR-134 [30]. Various RTKs, such as MET, EGFR, and PDGFR, all reduce the transcription of miR-134 upon binding to their ligands. Such inverse correlation is also observed in human GBM cells. Ectopic expression of miR-134 reveals its targets as KRAS and STAT5B, both of which are common downstream components of RTKs. By reducing the expression of KRAS and STAT5B, miR-134 reduces proliferation, invasion and tumor growth of GBM cells. Therefore, repressing miR-134 may represent a novel mechanism on how RTKs sustain signaling output in GBM cells.

The prevalence and complexity of MET/HGF signaling in high-grade GBM such as MET amplification, elevated HGF and pathway orchestration through intricate miRNA networks emphasize the importance of novel therapeutic strategies for targeting MET/HGF signaling for optimal therapy. With this in mind, we will discuss below the different therapeutic approaches being developed and discuss past and ongoing clinical trials targeting MET/HGF for therapy.

### 1.4. HGF/MET Deregulation in GBM

**Figure 3:**
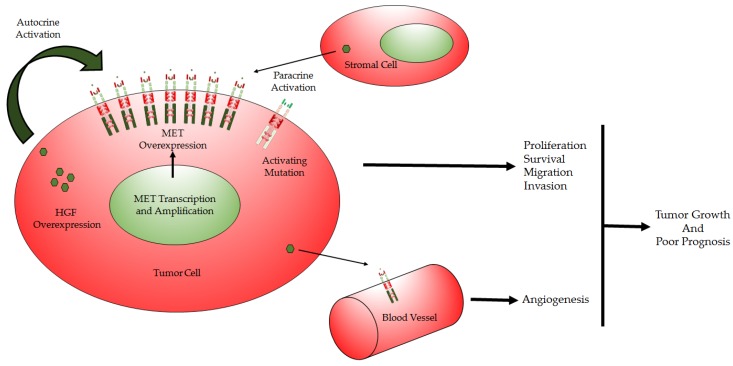
Modes of deregulation of HGF/MET in cancer: (1) Autocrine and paracrine overexpression of HGF, (2) Amplification of MET gene leading to MET protein overexpression, (3) An activating mutation resulting in a constitutively active protein product, (4) HGF overexpression by tumor cells activating MET in the tumor microenvironment.

Under normal conditions, HGF-induced MET activation is regulated by paracrine ligand delivery, ligand activation and ligand activated receptor internalization and degradation. Given the many molecular effectors that are known to enter into or result from MET recruitment, there are many mechanisms that lead to aberrant MET signaling including: overexpression of MET, elevated paracrine or autocrine ligand production, constitutive MET activation in the absence of MET gene amplification, and MET mutations [31]. Complications also arise from the fact that HGF/MET are highly expressed in GBM with expression correlating with grade [11]; indeed, this is compounded by co-expression of a HGF activator, a serine proteinase referred to as HGF activator (HGFA) [32], reinforcing an autocrine loop in highly malignant GBM [33]. Ultimately, the intrinsic feedback loop can amplify or promote the drivers of GBM through various mechanisms including inducing cell cycle progression, cell migration, invasion and angiogenesis and inhibition of apoptosis (Figure 3) [11].

### 1.5. HGF/MET Functions and Modes of Action in GBM

#### 1.5.1. Cell Cycle Regulation

A well-known mechanism of transformation is the ability of the cell to by-pass the critical cell cycle check point machinery and to advance into pro-growth processes; in particular, the main obstacle to clear is the G1 phase checkpoint, monitored by p53, where DNA integrity is assessed before replication. The p53 pathway is commonly dysregulated in GBM, where mutations or loss of p53 are common in secondary GBM and are present less frequently in primary GBM. Since p53 responds to DNA damage by inducing cell cycle arrest and apoptosis, loss or loss of function mutations of this tumor suppressor leads to deregulation of the cell cycle and enhanced tumor cell growth [34]. The HGF/MET signaling axis convenes with effectors on the p53 pathway through its regulation of p27, E2F-1 and c-Myc. HGF/MET not only prevents the accumulation of p27, a cyclin-dependent kinase (CDK) inhibitor, but also promotes its degradation allowing cell cycle progression in GBM cells, further driving malignancy. In addition, HGF can transcriptionally upregulate c-Myc, overexpression of which mediates G1/S transition irrespective of p27 [35]. HGF/MET’s involvement with E2F-1 also implicates its binding with RB, a well-known tumor suppressor. Under normal conditions, RB binds to and inhibits several transcription factors leading to cell cycle arrest and cell death; however, in GBM, loss of expression or mutation in RB leads to uncontrolled cell growth [34].

#### 1.5.2. Cell Proliferation and Evasion of Apoptosis

HGF induced activation of MET or mutations in the receptor that lead to constitutive activation stimulate the initiation of many downstream signaling pathways that promote tumor cell growth. The PI3K/Akt pathway, frequently altered in GBM, is deregulated through two mechanisms: aberrant activation of an RTK, such as MET, or deletion of the tumor suppressor PTEN. PTEN negatively regulates the PI3K/Akt pathway but is frequently lost or mutated in GBM. Up-regulation of the PI3K/Akt pathway in GBM leads to uncontrolled tumor cell growth and survival through aiding nuclear translocation of nuclear factor Κb (NFκB) which activates many cell survival and anti-apoptotic genes [36]. Moreover, through RAS activation, MET induces other cell survival signaling pathways such as MAPK, allowing tumor cells to grow, survive and evade apoptosis [34].

#### 1.5.3. Cell Migration and Invasion

Upon HGF stimulation of MET, brain tumor vascular cells express matrix metalloproteinases and urokinase, promoting matrix degradation and invasion [11]. Tumor cell invasion induced by MET is mediated through focal adhesion kinase (FAK) and STAT3 signaling [37]. FAK promotes tumor cell invasion and migration through both kinase-independent and kinase-dependent mechanisms; however, direct interaction of MET with FAK promotes HGF-induced cell motility and invasion in GBM [38,39]. STAT3 is also activated by MET and upregulation is a common attribute of GBM tumors where they act to modulate expression of genes involved in cell proliferation and invasion [40].

#### 1.5.4. Angiogenesis

HGF regulates angiogenesis through activation of MET on vascular endothelial cells, promoting vascular endothelial cell proliferation, migration, survival, and extracellular matrix degradation [11]. Previous research discovered that inhibition of MET significantly reduced tumor angiogenesis. MET is also implicated in epithelial-mesenchymal transition (EMT) in tumor endothelial cells correlating with increased plasticity and vessel malformation [41].

Interestingly, MET plays an important role in drug-acquired resistance. For example, in Bevacizumab sensitive GBM tumors, MET and vascular endothelial growth factor receptor 2 (VEGFR2) form complexes that suppress HGF mediated growth and invasion. However, in GBM tumors resistant to Bevacizumab, a VEGF-A neutralizing antibody, MET is further upregulated preventing this regulation and advancing invasion [37,42].

#### 1.5.5. Glioblastoma Stem Cells and Therapy Resistance

The cancer stem cell (CSC) model proposes an alternative view on tumorigenesis and cancer treatment. Drawing analogy from the normal stem cell biology, the CSC model states that the development and maintenance of a tumor is driven by a small population of CSCs within the tumor [43]. According to this model, therapies specifically targeting CSCs may prove to be effective in the context of conventional treatments. Research from the past three decades has provided ample evidence for the existence of CSCs in various type of tumors, including GBM [44]. Unfortunately, CSCs have been shown to be highly resistant to conventional chemotherapies or radiation therapies [45]. Therefore, identifying key signaling pathways that maintain the CSC population remains as the central focus of current research.

MET is a key signaling pathway in GBM stem cells (GSCs). Primary GBM cells with high MET expression contain higher number of GSCs and exhibit higher tumorigenicity [46]. Ectopic activation of MET in vitro is also capable of conferring a more GSC trait to GBM cells [47]. HGF, responsible for the self-renewal property of GSCs and MET a functional marker of GSCs, is present in approximately 40% of GSCs [48]. Using an elegant transposon mutagenesis approach, one group further demonstrates that MET activation is capable of directly transforming normal neural stem cells into GSCs, suggesting a possible route of GBM development [49]. Conversely, in GBM cells with high MET expression, inhibition of MET signaling reduces the number of GSCs within the population as well as their tumorigenicity [50,51,52]. The downstream signaling pathway of MET in GSC has not been fully explored. However, the Wnt/β-catenin pathway has been shown to at least facilitate the downstream effects of MET in GSCs, indicating a potential cross-talk between the two major pathways [53].

In addition to maintaining the GSC population, MET also plays a crucial role in conferring resistance to conventional therapies in GSCs. First, it has been known for many years that inhibiting MET enhances GBM responses to radiation [54,55,56]. Recently, a novel mechanism was identified where MET, surprisingly, is activated by radiation itself [57]. MET then activates the ATM pathway to initiate DNA repair, and also sequesters p21 to suppress apoptosis [48]. Both signaling arms dampen the effect of radiation therapy. Similarly, inhibiting MET signaling also provides a synergistic effect when co-administered with temozolomide (TMZ) [58], a commonly used alkylating agent in GBM patients. Perhaps the most interesting aspect of MET-conferred resistance comes from the cross-talk between MET and EGFR. Both receptors are commonly amplified in GBM, and both share similar downstream pathways [59]. Therefore, it should come as little surprise that MET activation can compensate for the lost signaling when EGFR is inhibited [60,61]. However, the cross-talk between MET and EGFR seem to be more complicated than simple compensation. Several groups have shown evidence that MET and EGFR are activated in different subset of tumors, each with distinct clinical course and survival outcome [52,62,63]. The relationship between the two subsets, if there any, has remained unclear until recently, when Jun et al. showed that inhibition of EGFR leads to activation of MET with increased level of stem cell traits [64]. One possible mechanism is that inhibition of wildtype EGFR leads to upregulation of a truncated version of EGFR known as EGFRvIII [65]. EGFRvIII is commonly present in GBM containing EGFR gene amplification and is capable of activating MET. The activated MET then upregulates its ligand HGF, thus forming an autocrine loop [66]. Whether this mechanism represents an evolution of GBM from EGFR-driven to MET-driven remains to be studied.

### 1.6. HGF/MET as GBM Therapeutic Targets

MET/HGF mutations and overexpression are present in a high percentage of GBM tumors and previous studies have demonstrated that inhibition of MET in tumor cells leads to growth inhibition, tumor regression and decreased metastatic potential. MET-amplification and overexpression has been reported in 5% and 13% of GBMs respectively, whereas MET gains are present in 47% of primary GBMs and 44% of secondary GBMs [67]. This finding is particularly effective for GBM cells that display high expression of both HGF and MET [14,17,68,69]. In that regard, targeting MET may prove beneficial for GBM therapy, where strategies to regulate the MET/HGF pathway have led to the development of several types of inhibitors: small-molecule tyrosine kinase inhibitors, monoclonal antibodies to the MET receptor and antagonists or monoclonal antibodies to HGF (Figure 2) [18].

#### 1.6.1. Competitors of MET (Preclinical)

Competitors of MET vie with the HGF ligand for binding to the receptor to prevent activation and subsequent initiation of downstream signalling pathways. NK2 and NK4, HGF antagonists, are intra-molecular fragments of HGF that compete for binding to the receptor to inhibit activity [68,69,70]. Although NK4 inhibits downstream pro-survival signalling, it also acts to inhibit angiogenesis whilst also enhancing radiation toxicity [70].

#### 1.6.2. Small-Molecule Inhibitors (Preclinical)

Due to the ubiquitous and prolific involvement of receptor tyrosine kinases in cancer pathology, many classes of small molecule inhibitors have been developed to combat RTKs through multiple modes of inhibition. Inhibitor types range from Types I–VI and are categorized by their binding patterns, kinetics and conformations, in addition to inhibiting multiple RTKs across different families [71]. Inhibitors for MET have primarily fallen under Types Ia, Ib and II in recent years, although it’s common for inhibitors from multiple classes to have interactions with RTKs related to their intended targets (in this case, MET), such as RON, VEGFR, and anaplastic lymphoma kinase (ALK). Inhibitors hailing from the Type I category are reversible, ATP-competitive inhibitors that are further divided into A and B subcategories, contingent on their modes of binding [68]. Ia members are selective, u-shaped inhibitors that bind the active conformation of their RTK target within the ATP binding pocket in addition to the hydrophobic pocket [68,71]. Conversely, Ib members are known to bind the inactive conformation of their target within the ATP binding pocket but do not reach the hydrophobic pocket. Despite these differences, both Ia and Ib inhibitors feature robust selectivity [68]. One preclinical example that features type I attributes is the oral compound AMG 337 [72]. AMG 337 is ATP-competitive and has shown selectivity for the inactive activation loop of MET; the selectivity was further demonstrated through competitive binding assays of 402 kinases. In vitro, the GBM cell line U87 MG showed partial sensitivity to AMG 337, although the nature of the partial sensitivity can probably be correlated with U87’s autocrine HGF secretion [72]. Nevertheless, AMG 337 showed limited toxicity in mouse xenograft models and was reasonably tolerated.

Advances in genotyping and centralized tissue bank systems (for example, The Cancer Genome Atlas, also known as TCGA) have allowed for the cataloguing and categorization of genetic alterations such as gene amplification, mutation, deletions and other modifications implicated in multiple cancer types; many of these alterations happen, unsurprisingly, in RTKs. Therefore, when designing small-molecule inhibitors for RTKs, it is imperative to determine the rates of amplification, mutation and deletions within a given cancer and to avoid potential modes for resistance. Berthou and colleagues show one mode of this strategy, where they demonstrated the compound SU11274 targets wild type MET and two mutants, M1268T and H1112Y in stably expressed NIH 3T3 cells [73]. This multi-nodal RTK approach would be best suited to treating cancers with high levels of MET mutation, such as hepatocellular and gastric carcinomas. Other investigators are following suit to accommodate mutations and compensation through parallel RTK pathways by choosing to focus on type II inhibitors [74]. Type II inhibitors, like their type I counterparts, are also ATP-competitive but have been shown to have much more variable features that lead to a more diverse RTK binding profile [68]. In line with the work conducted by Norman and colleagues, structure-activity relationship (SAR) studies in the follow up paper released by Liu et al. show that type II inhibitor selectivity for specific RTK members can be improved significantly—ultimately, these SAR studies yielded the inhibitor AMG 458 [75]. Taking this further, some investigators have chosen to focus on developing type II inhibitors for specific modes of resistance in cancer models, such as NVP-BVU972 in conjunction with BCR/ABL point mutations in chronic myeloid leukaemia (CML) [76] and NPS-1034 in EGFR inhibitor resistant lung cancer models [77]. Further still, another mode would be to develop a small molecule inhibitor to work in combination with an already approved inhibitor, such as what Burbridge and colleagues have done with S49076 and combination studies with bevacizumab [78]. This strategy of exploiting a less-selective type II RTK binding profile may have merit in GBM, where EGFR is predominately amplified and has been shown to propagate cancer signalling in parallel to MET [79].

Alongside of type I and II ATP-competitive inhibitors, other inhibitors can maintain inhibition modes outside of ATP competition, such as allosteric inhibitors and covalent inhibitors (types III through types VI) [71].

#### 1.6.3. Small-Molecule Inhibitors (Clinical)

One common type Ia member is the MET/ALK inhibitor crizotinib/PF-02341066, a small molecule inhibitor developed by Pfizer. Crizotinib has been shown to effectively inhibit MET-dependent growth, invasion and survival [69] and is currently in phase II clinical trials for CNS and solid brain tumours (Table 1). Crizotinib has also displayed significant anticancer effects in non-small cell lung cancer (NSCLC) with limited overall toxicity and recently completed a phase III clinical trial for NSCLC patients with ALK rearrangement [80]. Similar to crizotinib, cabozantinib/XL184 (Exelixis), is an oral type I inhibitor of MET, but its targets also include VEGF and AXL and has already been approved for the treatment of metastatic medullary thyroid cancer [81,82]. In GBM tumour models, cabozantinib has been shown to inhibit phosphorylation of MET resulting in decreased proliferation, invasion and activation of apoptosis [83]; due to its preclinical success, it recently completed a phase II trial for adult GBM patients (Table 1). Another ATP-competitive MET inhibitor, capmatinib/ INCB28060, has completed phase I trials for multiple cancers and is currently underway in a phase I trial in GBM patients (Table 1) [84]. The first non-ATP-competitive inhibitor of MET, ARQ197/Tivantinib (ArQule), acts to prevent HGF-stimulated MET phosphorylation thus inhibiting activation of downstream survival pathways such as PI3K, MAPK and STAT3. Tivantinib is currently in phase I trials for patients with metastasis and is proving to be well tolerated with several reports of stable disease [69].

Other small molecule inhibitors currently being investigated in other forms of cancer, excluding GBM, include: Foretinib/GSK1363089 (Exelixis), an ATP-competitive inhibitor of MET and VEGFR2, has been investigated clinically in phase I and II trials in many cancer types including head and neck, NSCLC, breast, liver and metastatic gastric cancer with varying results; Amuvatinib/MP470 (SuperGen), an inhibitor of MET, c-Kit, PDGFRα and Flt-3, has completed phase I and II trials in multiple tumor types including small cell lung cancer, solid tumors and lymphoma [69]; MK-2461, an inhibitor of MET, FGFR1/2/3 and Flt-1/3/4, has completed phase II trials in neoplasms; Glesatinib/MGCD-265 (Mirati Therapeutics), an ATP-competitive inhibitor of MET and ALK, has completed a phase II clinical trial for NSCLC patients with driver mutations in MET indicating partial responses and even tumor regression and Tepotinib, a selective MET inhibitor, has demonstrated promising anti-tumor activity in a phase I clinical trial for solid tumors and a phase II trial for advanced lung cancer is currently enrolling [85].

#### 1.6.4. Monoclonal Antibodies against MET and HGF

Monoclonal antibodies act to obstruct binding of HGF to the MET receptor preventing activation of downstream pathways leading to growth inhibition, activation of apoptosis and inhibition of metastasis. One major drawback to using monoclonal antibodies for cancer therapies is the decreased penetration of the blood-brain barrier observed compared to small molecule inhibitors. This issue is addressed with the use of novel delivery techniques [69,86].

AMG102/Rilotumumab (Amgen, Inc., Thousand Oaks, CA USA), a human IgG2 monoclonal antibody, binds to MET and inhibits the binding of HGF to MET. AMG102 has completed phase I and II clinical trials in GBM patients with reports of partial response and stable disease (Table 1) [87]. One potential concern with this trial is that enrollment was not in line with the trial stipulations. Combining this therapy with currently used chemotherapeutics such as bevacizumab or TMZ has also shown to be effective in achieving stable disease [88]. MetMAb/Onartuzumab (Genentech), a monovalent, monoclonal anti-MET antibody, binds to MET to prevent binding of HGF thus blocking activation of downstream signaling pathways. MetMAb was designed as a monovalent antibody to avoid agonistic activity that sometimes occurs when a divalent antibody is used [89]. Preclinical studies of MetMAb demonstrated its ability to inhibit MET phosphorylation and therefore inhibit downstream pro-survival pathways in GBM cell lines. MetMAb, in combination with bevacizumab, completed a phase II clinical trial for patients with recurrent GBM (Table 1). Unfortunately, a phase III clinical trial in patients with NSCLC failed to demonstrated effective therapy [69,89].

## 2. Conclusions and Future Perspectives

Inhibition of MET has demonstrated clinical benefit for patients with multiple types of cancer. In addition to showing that MET inhibitors are generally well-tolerated, previous clinical trials (Table 1) have also highlighted that patient selection is an important consideration. A specific subset of patients, those that exhibit MET gene amplification, respond more successfully to MET targeted therapy emphasizing that clinical trials should be tailored to this subset of patients to achieve maximum therapeutic outcome. Additionally, we and others have established that autocrine production of HGF is a predictor of response to MET inhibition [14,90,91]. Furthermore, combining MET inhibitors with other RTK inhibitors, such as EGFR, may further enhance anticancer activity [90]. In the event that MET inhibitors alone are insufficient, they reportedly still act to enhance radiation and chemotherapeutic toxicity in a large cohort of patients, as well as reversing acquired resistance to other RTK inhibitors [8,10,17].

Furthermore, therapeutic regulation of miRNAs in GBM tumors will likely lead to increased survival for patients. In the past, lack of an efficient delivery system for miRNAs, especially into the central nervous system, was the main hurdle. In recent years, various delivery mechanisms have been investigated, for example, miRNAs can be conjugated to modified gold nanoparticles, which are able to carry miRNAs across the blood-brain barrier, as in the case of miR-182 [29]. Gold nanoparticles also demonstrate low toxicity, high stability and high cell uptake, making them the most promising delivery tool for miRNAs [92]. Other delivery mechanisms available include, adeno-associated virus [93], polymeric brain-penetrating nano-particles [94] and mesenchymal stem cells [95,96]. Both of which are capable of reliably transport miRNAs and have demonstrated brain-specific tropism. However, no studies to date have demonstrated in vivo effects against GBM using either of these miRNA carriers.

HGF/MET inhibitors, specifically small molecule inhibitors and monoclonal antibodies, have displayed mixed results in clinical trials in GBM patients that emphasize the importance of patient selection. Combining HGF/MET inhibitors with currently used chemotherapeutics or inhibitors for other growth pathways would prove to be a more effective treatment options for many cancer patients.

## Figures and Tables

**Table 1:** Overview of previous and current MET/HGF inhibitor clinical trials.

Clinical Trial	Phase	Status	Drug	Combinations	Patient Population	Results
NCT 02386826	Ib	Recruiting	INCB28060 (INC280)	Bevacizumab	Recurrent GBM, Metastatic Colorectal Cancer (mCRC) Metastatic Renal Cell Carcinoma (mRCC)	None Reported
NCT 01870726	II	Completed	INCB28060 (INC280)	Buparlisib	Recurrent GBM	None reported
NCT 01441388	Ib	Withdrawn	Crizotinib	VEGF inhibitors, axitinib, sunitinib, bevacizumab and sorafenib	Advanced solid tumors in GBM, Renal Cell Carcinoma (RCC) and Hepatocellular Carcinoma (HCC)	None Reported
NCT 00939770	I/II	Active, not recruiting	Crizotinib	-	Relapsed/refractory solid tumors in brain and central nervous system, neuroblastoma and anaplastic large cell lymphoma	None Reported
NCT 01644773	I	Recruiting	Crizotinib	Dasatinib	High-grade glioma, diffuse intrinsic pontine glioma	None Reported
NCT 02034981	II	Recruiting	Crizotinib	-	MET amplified GBM	None Reported
NCT 01632228	II	Completed	Onartuzumab	Onartuzumab with bevacizumab versus bevacizumab alone or onartuzumab monotherapy	Recurrent GBM	None reported
NCT 01113398	II	Completed	Rilotumumab	Bevacizumab	Recurrent malignant glioma	16.67% of cohort experienced serious adverse events
NCT 00427440	II	Completed	Rilotumumab	-	Advanced malignant glioma	None reported
NCT 00960492	I	Completed	Cabozantinib	Temozolomide, radiation therapy	GBM, Giant Cell GBM and Gliosarcoma	None reported
NCT 00704288	II	Completed	Cabozantinib	-	Recurrent GBM	None reported
NCT 01189513	I	Withdrawn	Ficlatuzumab	-	GBM	None Reported
NCT 01433991	I/II	Active, not recruiting	Golvatinib	Lenvatinib	Recurrent GBM, unresectable stage III/IV melanoma	None Reported
